# Hypersensitive blood vessels in Clarkson disease

**DOI:** 10.1172/JCI180795

**Published:** 2024-05-15

**Authors:** Emmanuel Nwadozi, Lena Claesson-Welsh

**Affiliations:** Department of Immunology, Genetics and Pathology, Rudbeck Laboratory, Uppsala University, Uppsala, Sweden.

## Abstract

Idiopathic systemic capillary leak syndrome (ISCLS) is a rare, recurrent condition with dramatically increased blood vessel permeability and, therefore, induction of systemic edema, which may lead to organ damage and death. In this issue of the *JCI*, Ablooglu et al. showed that ISCLS vessels were hypersensitive to agents known to increase vascular permeability, using human biopsies, cell culture, and mouse models. Several endothelium-specific proteins that regulate endothelial junctions were dysregulated and thereby compromised the vascular barrier. These findings suggest that endothelium-intrinsic dysregulation underlies hyperpermeability and implicate the cytoplasmic serine/threonine protein phosphatase 2A (PP2A) as a potential drug target for the treatment of ISCLS.

## Idiopathic systemic capillary leak syndrome

Massive vascular permeability leading to systemic edema, hypoproteinemia, and hypotensive shock, denoted capillary leak syndrome (CLS), can be a sequela to sepsis, trauma, or other severe conditions (1). A distinct subgroup named idiopathic systemic CLS (ISCLS), or Clarkson syndrome, was identified by Bayard D. Clarkson in the 1960s. ISCLS is a rare condition that has thus far been identified in approximately 500 individuals worldwide (2), mostly middle-aged adults of both sexes. ISCLS stands out, as there are no known triggers for the recurrent, dramatic episodes of acute hyperpermeability (also known as flares).

ISCLS flares are triggered by upper respiratory tract infections, e.g., from influenza virus (2), but a direct cause-consequence relationship has not been established. After the acute phase of the flare, the condition spontaneously recedes or deteriorates to acute kidney failure, rhabdomyolysis, and disseminated intravascular coagulation. In approximately 30% of cases, patients die (3). Given the similarity of symptoms to other CLS conditions, the dramatic course of flares, and the rarity of patients, ISCLS remains challenging to diagnose and treat. In fact, the most efficient therapy, apart from fluid replacement, appears to be prophylactic intravenous administration of immunoglobulins, although it is unclear why this treatment is beneficial (4). Another poorly understood feature of ISCLS is the presence of monoclonal γ-globulins in the majority of patients’ serum (2).

The etiology of ISCLS has remained an enigma. There is no familial aggregation (5) or common genetic insult (6) identified as yet. A potential susceptibility locus at chromosomal region 3p25.3 includes the *ARHGAP5* gene (7). In one case of childhood ISCLS, a mutation in the corresponding protein p190BRho GTPase–activating protein (GAP) was identified and shown to be associated with vascular hyperpermeability in cell cultures (8). However, not all patients with ISCLS carry mutations in the *ARHGAP5* gene. A lack of clear-cut diagnostic tools such as genetic tests has contributed to an underdiagnosis of ISCLS. As such, better understanding of how vascular hyperpermeability is established in ISCLS, as well as the development of diagnostic tests and new therapies, is much needed.

## Vascular hypersensitivity in ISCLS

Normally, vessels are sealed and do not permit the passage of plasma proteins or cells into tissues. This vascular barrier is composed of endothelial cells lining the vessel wall, the surrounding basement membrane, and supporting mural cells. During ISCLS flares, the vascular barrier is destroyed.

**Figure 1:**
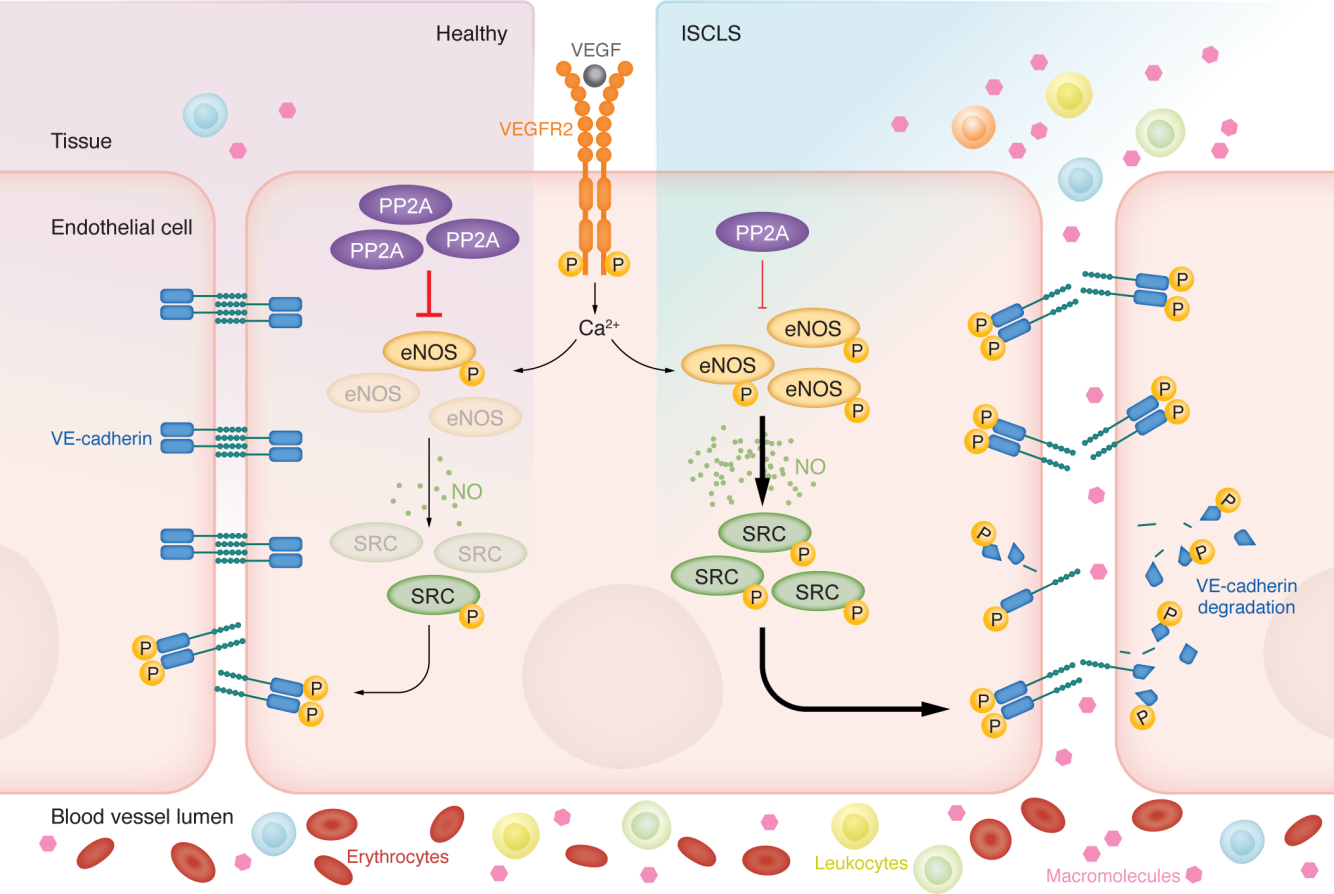
Endothelial cells show hypersensitivity in ISCLS. VEGF binding to VEGFR2 initiates a cascade of intracellular events involving calcium (Ca^2+^) release, eNOS phosphorylation at S1177, NO production, and activating nitration of Src. Subsequently, phosphorylation of VE-cadherin at Y685 results in the disruption of VE-cadherin homophilic interactions, leading to cellular and macromolecular leakage. In ISCLS, reduced levels of the cytoplasmic serine/threonine protein PP2A renders the vasculature hypersensitive to agonists such as VEGFA, resulting in exacerbated leakage.

Two main mechanisms prevail in regulating the passage of blood constituents across the vessel wall: the transcellular and the paracellular routes (9). Fluid and small molecules can diffuse through the vessel wall, driven by the hydrostatic pressure. Passage of large molecules and cells, however, requires the opening of junctions between endothelial cells. Endothelial junctions are organized in tight and adherens junctions. While tight junctions are regarded as gate keepers of vascular wall integrity, for example in the central nervous system, less is known about their contribution to increased permeability in the living organism. In contrast, as shown in numerous in vivo studies in mice, transient, reversible disruption of adherens junctions is required for the passage of molecules and cells from blood into tissues (9) (Figure 1). Adherens junctions are composed of the transmembrane protein vascular endothelial–cadherin (VE-cadherin), which bridges adjacent endothelial cells in homophilic complexes, keeping the junctions sealed. When endothelial cells are exposed to leakage agonists such as VEGF or inflammatory cytokines such as histamine or bradykinin, VE-cadherin becomes tyrosine phosphorylated (e.g. at residue Y685) and internalized. Thereby, partially disrupted adherens junctions create discrete gaps between endothelial cells. Src family kinases (SFKs) mediate the phosphorylation of VE-cadherin that triggers its internalization (10). VE-cadherin endocytosis is also affected by blood flow–regulated mechanotransduction. Once internalized, VE-cadherin may be degraded or recycled back to the cell surface to re-seal the junction.

In the healthy condition, only specific segments of the vasculature, namely venous capillaries and small veins, permit leakage of molecules and cells. Moreover, few endothelial junctions in these vessel segments form gaps in response to leakage agonists (11). In contrast, in ISCLS, most endothelial junctions are likely engaged, resulting in massive efflux of plasma constituents into tissues. Still, ISCLS-engaged vessels are morphologically normal (2). Known permeability agonists, such as VEGF and angiopoietin 2, are elevated in serum from patients during flares compared with those in remission (12), but therapies directed toward reducing the function of these molecules such as anti-VEGF neutralizing antibodies, have not been clinically successful (2). Thus, the ISCLS enigma is not explained by increased production of circulating agonists. Instead, the ISCLS vasculature may be hypersensitive to leakage agonists.

Indeed, the involvement of vascular hypersensitivity in the etiology of ISCLS is demonstrated in the study by Ablooglu et al. (13) in this issue of the *JCI*. The authors used a range of patient-derived biopsies, skin and blood outgrowth endothelial cells (BOECs), and a hypersensitive mouse model (SJL/J) to arrive at several conclusions. (a) Skin vessels from patients with ISCLS showed increased levels of VE-cadherin phosphorylated on Y685. (b) There were increased levels of endothelial nitric oxide synthase (eNOS) phosphorylated on S1177, in BOECs. (c) When eNOS activity was inhibited pharmacologically in SJL/J mice, leakage was efficiently suppressed. (d) Expression of the A subunit of the cytoplasmic serine/threonine protein phosphatase 2A (PP2A) was reduced in BOECs (Figure 1). The dysregulation of VE-cadherin in ISCLS agrees with data reported by Wu et al. (14), who used a fibrin-derived, VE-cadherin binding peptide, FX06, to stabilize endothelial junctions, which normalized VE-cadherin morphology.

## Molecular mechanisms

Why is VE-cadherin phosphorylation dysregulated in ISCLS endothelial cells? Phosphorylation of VE-cadherin by Src is dependent on activation of Src tyrosine kinase activity. Full Src activation occurs in several steps, including eNOS-derived, NO-dependent tyrosine nitration of Src (15). ISCLS hypersensitivity would therefore be explained by increased levels of activated phosphorylated S1177 (p-S1177) eNOS, promoting accumulation of active Src, which, in turn, more vigorously phosphorylates VE-cadherin. The next obvious question is: why are p-S1177 eNOS levels increased? A possible reason involves the reduction in PP2A expression, as shown by Ablooglu et al. (13). The demonstration that PP2A overexpression stabilizes endothelial junctions in BOEC cultures strengthens the evidence for its involvement in the pathophysiology of ISCLS. PP2A has been shown to dephosphorylate eNOS (16), however, PP2A is ubiquitously expressed and has hundreds of other substrates, some of which are implicated in cell proliferation. PP2A has, moreover, been referred to as a tumor suppressor, and expression levels are reduced in several human cancer forms (17). Structurally, PP2A is assembled from three subunits: the structural A chain, the regulatory B chain, and the catalytic C chain; and for each chain, there are several subunits. In ISCLS, only expression for the A chain was reduced. In accordance, Ablooglu et al. found a potentially deleterious SNP in the PP2A A chain (*PPP2R1B*), but not in the other chains. This genetic alteration may affect assembly and function of the enzyme. There are several SNPs also for eNOS, and these variants may explain why eNOS appears in aggregates in ISCLS endothelial cells, as reported by Ablooglu et al. (13).

## Future perspectives

Detailed studies are needed to understand how eNOS and PP2A contribute to the establishment of ISCLS. Both enzymes play multiple roles in the endothelium. In addition to its role in Src activation, eNOS/NO also regulates vascular tone through the relaxing effect of NO on vascular smooth muscle cells (18). eNOS inhibitors are available, and Ablooglu et al. showed that eNOS inhibition using *N*(γ)-nitrol-l-arginine methyl ester (l-NAME) in the hypersensitive mouse model SJL/J suppressed vascular leakage. PP2A is a complex drug target for which enzymatic activity would need to be restored, not inhibited. Interestingly, small-molecule drugs that stabilize and increase the activity of PP2A in an isotype-specific manner are being developed for cancer therapy (19). There may be opportunities for therapeutic development and application of such drugs also in ISCLS.

With more patients now being correctly diagnosed with ISCLS, larger biobanks can be established to allow in-depth genetic analyses. Thereby, it should be possible to ultimately identify one or several key genetic insults leading to ISCLS and, importantly, determine their role in triggering ISCLS flares.

## Version 1 05/15/2024

Electronic publication
